# The RNA-binding protein Nab2 regulates the proteome of the developing *Drosophila* brain

**DOI:** 10.1016/j.jbc.2021.100877

**Published:** 2021-06-15

**Authors:** Edwin B. Corgiat, Sara M. List, J. Christopher Rounds, Anita H. Corbett, Kenneth H. Moberg

**Affiliations:** 1 Department of Cell Biology, Emory University School of Medicine, Emory University, Atlanta, Georgia, USA; 2 Graduate Program in Genetics and Molecular Biology, Emory University, Atlanta, Georgia, USA; 3 Department of Biology, Emory University, Atlanta, Georgia, USA; 4 Graduate Program in Neuroscience, Emory University, Atlanta, Georgia, USA

**Keywords:** Nab2, polyadenosine RNA-binding protein, intellectual disability, neuronal morphology, global proteomics, APF, after puparium formation, GO, gene ontology, MBs, mushroom bodies, MEME, Multiple Expectation maximizations for Motif Elicitation, PCA, principal component analysis, PCP, planar cell polarity, RBPs, RNA-binding proteins

## Abstract

The human *ZC3H14* gene, which encodes a ubiquitously expressed polyadenosine zinc finger RNA-binding protein, is mutated in an inherited form of autosomal recessive, nonsyndromic intellectual disability. To gain insight into neurological functions of ZC3H14, we previously developed a *Drosophila melanogaster* model of ZC3H14 loss by deleting the fly ortholog, Nab2. Studies in this invertebrate model revealed that Nab2 controls final patterns of neuron projection within fully developed adult brains, but the role of Nab2 during development of the *Drosophila* brain is not known. Here, we identify roles for Nab2 in controlling the dynamic growth of axons in the developing brain mushroom bodies, which support olfactory learning and memory, and regulating abundance of a small fraction of the total brain proteome. The group of Nab2-regulated brain proteins, identified by quantitative proteomic analysis, includes the microtubule-binding protein Futsch, the neuronal Ig-family transmembrane protein turtle, the glial:neuron adhesion protein contactin, the Rac GTPase-activating protein tumbleweed, and the planar cell polarity factor Van Gogh, which collectively link Nab2 to the processes of brain morphogenesis, neuroblast proliferation, circadian sleep/wake cycles, and synaptic development. Overall, these data indicate that Nab2 controls the abundance of a subset of brain proteins during the active process of wiring the pupal brain mushroom body and thus provide a window into potentially conserved functions of the Nab2/ZC3H14 RNA-binding proteins in neurodevelopment.

Neurons develop complex architectures that allow them to function within massive interconnected networks that transmit electrochemical signals among thousands of other neurons in a shared circuit. The polarized morphology of neurons is particularly unique, with each cell containing axons and dendrite projections that can extend over enormous distances relative to the size of the cell body. Axonal growth and guidance is largely directed through the growth cone, which responds to guidance cues to steer the axon (1, 2). This axonal guidance is regulated in part by local translation of mRNAs within the growth cone that modifies the local proteome. This process of local translation, which relies on predelivery of mRNAs to the axon tip, facilitates rapid shifts in translation in response to extracellular cues that would otherwise be limited by distance from the nucleus and relatively slow speed of intracellular transport (1, 2, 3, 4). The local translation of mRNAs in distal neuronal projections is critical for proper development of the nervous system (1, 3) but poses many biological challenges, including the need to maintain mRNAs in a translationally repressed state during transport from the nuclear periphery to distal sites where regulated translation must occur (2, 4). RNA-binding proteins (RBPs) play a major role in this process (4).

RBPs play critical roles in regulating temporal and spatial expression of numerous mRNAs that encode proteins with roles in neuronal function (5). Although RBPs play broadly important roles in regulating multiple steps in gene expression shared by all cell types, mutations in genes encoding RBPs often result in tissue type– or cell type–specific diseases (2, 4, 6, 7, 8, 9, 10). A large number of these RBP-linked diseases include significant neurologic impairments, which likely reflects an enhanced reliance on post-transcriptional mechanisms to pattern spatiotemporal gene expression over the long distances that neurons extend (1, 11, 12). This dependence on RBP-based mechanisms of gene expression is exemplified by disease-causing mutations in the genes (4) encoding the fragile X mental retardation protein (13), survival of motor neuron protein (14), and TAR DNA-binding protein 43 (11). Mutations in the *ZC3H14* gene, which encodes a zinc finger RBP (zinc finger CysCysCysHis-type 14), cause neurological defects that broadly resemble those associated with these more extensively characterized RBPs (4, 15).

The human *ZC3H14* gene encodes a ubiquitously expressed polyadenosine RBP that is lost in a heritable nonsyndromic form of intellectual disability (15). The *Drosophila* ZC3H14 homolog, Nab2, has provided an excellent model to probe the function of ZC3H14/Nab2 in neurons (16, 17, 18). *Nab2* deletion in flies results in defects in locomotion and neuromorphology that are rescued by neuron-specific re-expression of Nab2 (17). Neuron-specific expression of human ZC3H14 partially rescues many of the Nab2 null phenotypes, demonstrating a high level of functional conservation between ZC3H14 and Nab2 (17, 18, 19).

Nab2 and its orthologs are found primarily in the nucleus at the steady state (20, 21, 22, 23, 24, 25), but evidence shows that these proteins can shuttle between the nucleus and cytoplasm (22, 23, 26). Within neurons, small pools of Nab2 are detected within axons and dendrites (15, 16, 17, 20, 21), raising the possibility that Nab2 has both nuclear and cytoplasmic roles in this cell type. Multiple studies in a variety of model organisms have defined key roles for Nab2 in pre-mRNA processing events within the nucleus, including regulation of splicing events (19, 27, 28), transcript termination (19, 29), and control of poly(A) tail length (16, 19, 27, 28, 29). Additional studies localize Nab2 within cytoplasmic mRNA ribonucleoprotein particles and imply roles in translational repression, likely mediated in part through interactions with fragile X mental retardation protein (20, 21, 22, 27). Ultimately, all of these post-transcriptional regulatory events are likely to alter levels of key proteins that are critical for proper neuronal function.

At a morphological level, zygotic deficiency for Nab2 produces structural defects in the adult *Drosophila* brain mushroom bodies (MBs) (17), twin neuropil structures that mirror across the brain midline and are required for olfactory learning and memory (17, 30, 31). The MBs are formed of five lobes: γ, α, α’, β, and β’ (Fig. 1*A*) (32, 33). In the fully formed adult brain, *Nab2* null neurons fail to project axons into the α-lobe and β-lobe axons inappropriately cross the midline into the contralateral hemisphere (17, 20). These findings implicate Nab2 in developmental control of axonogenesis and growth cone guidance. MB development begins in the larval stage with neuroblast pools that project axons into nascent γ-lobes (32, 33, 34, 35, 36). During the subsequent pupal stage, these γ-lobes are pruned back, and α- and β-axons begin to project into their corresponding tracks (32, 33, 34, 35, 36). By 24 h after puparium formation (APF), α- and β-lobes have formed their initial structure and are being thickened by new axons that project through the core of the bundle. This process continues through ∼72 h APF, when the α- and β-lobes are fully formed (32, 34). The effect of *Nab2* alleles on final α- and β-lobe structure in the adult brain implies a role for the Nab2 RBP in axon projection and guidance during early pupal stages (17, 20, 33, 34, 35).

**Figure 1:**
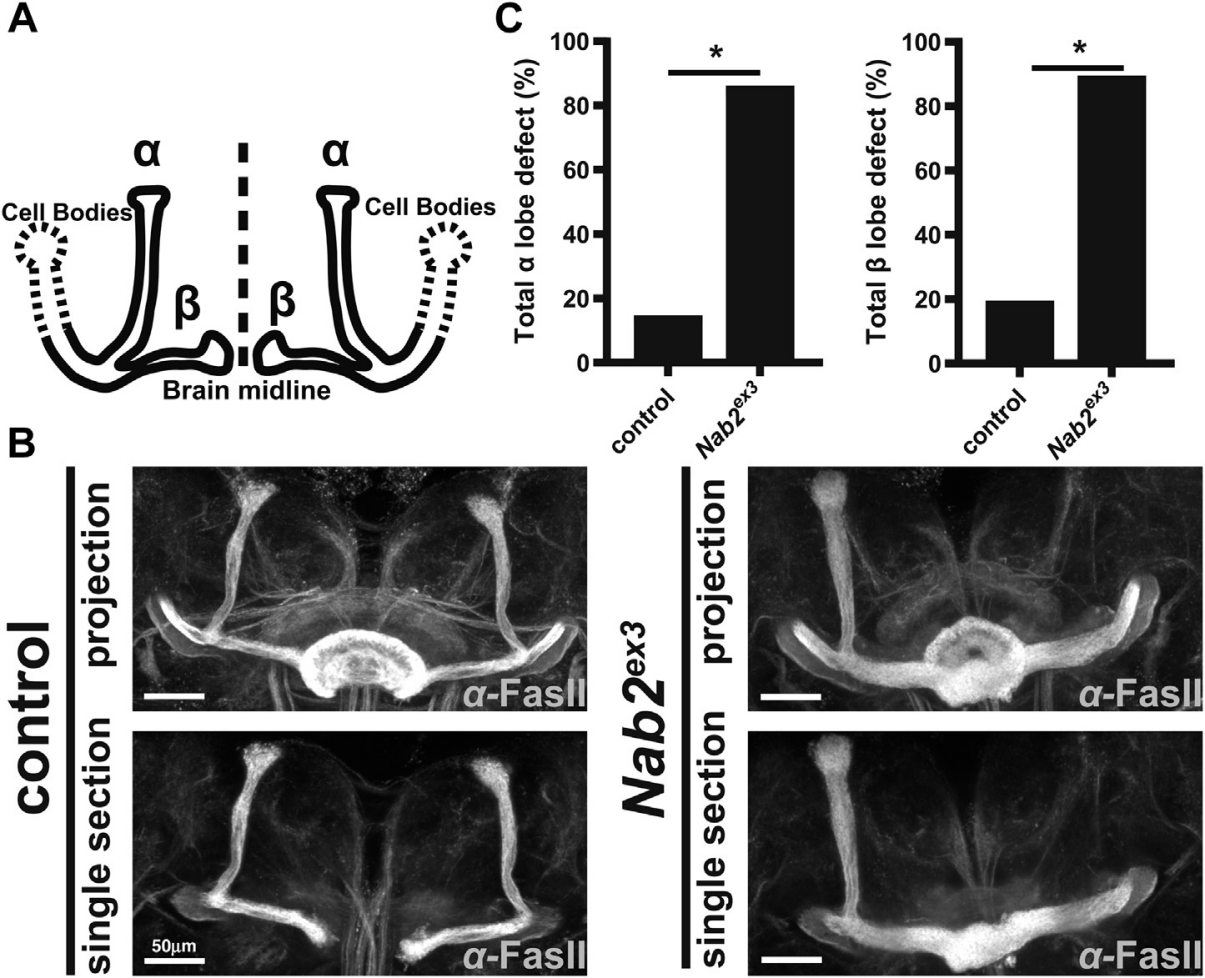
**Nab2 is required during pupal development for proper neuromorphological patterning of the mushroom bodies.***A*, diagram of the *Drosophila* mushroom body depicting cell bodies (*dashed lines*) projecting axons that bundle to make the dorsal (α) and medial (β) lobes that are mirrored across the brain midline (*dashed line*). *B*, Fasciclin II (FasII) antibody staining of *control* (*C155>Gal4, w*^*1118*^) and *Nab2*^*ex3*^ (*C155>Gal4;;Nab2*^*ex3*^) brains 48 to 72 h after puparium formation. Confocal images show maximum intensity Z-stack projections (projection) that display full mushroom bodies and single transverse plane sections (single section) that display midline crossing of β-lobe axons. Imaging reveals that control rarely shows defects in α- and β-lobes, while *Nab2*^*ex3*^ brains often have thinning or loss of the α-lobes and β-lobes that project across the midline into the contralateral hemisphere resulting in the fusion of the lobes or occasionally loss of β-lobes. The ellipsoid body (*donut*-shaped structure at the brain midline) is visible in maximum intensity projection images that mask the β-lobe status, so single section images are included for clarity. *C*, quantification of the frequency of control and *Nab2*^*ex3*^ (*left*) total α-lobe defect (thinning or missing α-lobe) or (*right*) total β-lobe defect (fusion or missing β-lobes) using the scoring system as described in Experimental procedures. Control (α-lobe = 11 biological and 22 technical replicates; β-lobe = 11 biological and technical replicates) and *Nab2*^*ex3*^ (α-lobe = 17 biological and 34 technical replicates; β-lobe = 17 biological and technical replicates). The *asterisk* indicates *p* < 0.05; α-lobe *p* = 0.002; β-lobe *p* = 0.007.

Here, we exploit the predictable time course of brain development in *Drosophila* to perform temporally coupled analysis of the effect of Nab2 loss on the pupal brain proteome and the process of axon projection into the forming pupal MBs. We find that Nab2 loss disrupts α- and β-axon projection in the pupal MBs coincident with significant increases in the steady-state abundance of proteins that are enriched for roles in neurodevelopment, neuronal and glial metabolism, axon guidance, and trans-synaptic signaling. Complementary analysis of neuron-specific Nab2-overexpressing brains confirms that a subset of these proteins also change abundance in response to excess Nab2. In sum, this paired morphological-proteomic analysis provides strong evidence that Nab2 is required to control the abundance of proteins with critical roles in *Drosophila* neurons that may play conserved roles in humans.

## Results

### Nab2 loss disrupts axon projection into the forming pupal MBs

Our prior finding that loss of Nab2 impairs MB neuromorphology in the mature adult *Drosophila* brain (4, 6, 7) suggests a role for Nab2 in MB morphogenesis in the preceding pupal phase. Consistent with this idea, serial optical sectioning of α-FasII-stained *Nab2*^*ex3*^ (*i.e.*, zygotic null) and control brains 48 to 72 h APF reveals thinning or missing α-lobes and β-lobes that project and fuse across the midline that are not present to the same extent in control brains (Fig. 1, *A* and *B*). The 48- to 72-h APF time window coincides with a midpoint in projection and guidance of α- and β-lobes. At this stage, control brains show incompletely formed α- and β-lobes with a low degree of defects (13% and 18%, respectively), whereas *Nab2*^*ex3*^ brains already display a high rate of missing/thinning α-lobes and fused/missing β-lobes (both 85%) (Fig. 1*C*). These data indicate that *Nab2* is required during pupal projection and guidance of the MB axons, raising the question of how loss of the Nab2 RBP affects the pupal brain proteome.

### Quantitative proteomic analysis of developmentally timed pupal brains

**Figure 2:**
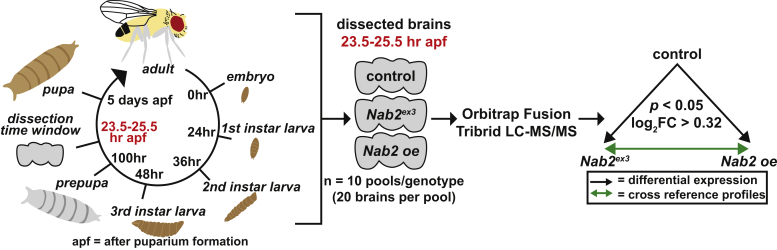
**Study design and analytic approach for quantitative proteomic analysis of *Drosophila* pupal brains.** A workflow summary showing dissection window, experimental design, and analysis. The *Drosophila* life cycle with developmental stage and hours of development depicted with the dissection time window (23.25–25.5 h APF) in *red*, *left*. There were 600 developmentally timed brain samples that were pooled by the genotype, control (*C155>Gal4, w*^*1118*^); Nab2 zygotic null (*Nab2*^*ex3*^ = *C155>Gal4;;Nab2*^*ex3*^); and *Nab2 overexpression in neurons* (*Nab2 oe* = *C155>Gal4;Nab2*^*EP3716*^*;Nab2*^*ex3*^), and by sex, resulting in 30 individual pools, center. Each sample pool was processed, analyzed using an Orbitrap Fusion Tribrid Mass Spectrometer and quantified using MaxQuant against the *Drosophila melanogaster* UniProt database, center. *Arrows* depict the performed analyses. Differential protein abundance of *Nab2*^*ex3*^ and *Nab2 oe* brains was calculated with an FDR-adjusted *p*-value (*black arrows*) and then second-degree analyses cross-referencing the *Nab2*^*ex3*^ and *Nab2 oe* proteomic profiles (*green arrows*), *right*. APF, after puparium formation; FDR, false discovery rate.

Nab2 has been identified as a component of cytoplasmic ribonucleoprotein particles linked to mRNA trafficking and translation (20, 23, 25) and as a nuclear component of post-transcriptional complexes (20, 21) that control mRNA splicing (22, 27, 28), transcription termination (29), and polyadenylation (16). To explore how *Drosophila* Nab2 affects the mRNA-derived proteome in the developing pupal brain, global label-free LC-MS/MS was performed on dissected 24-h APF brains of control (*C155>Gal4, w*^*1118*^), mutant *Nab2*^*ex3*^ (*C155>Gal4;;Nab2*^*ex3*^), and neuron-specific *Nab2* overexpression (*Nab2 oe*) (*C155>Gal4;Nab2*^*EP3716*^*;Nab2*^*ex3*^) animals as illustrated in Figure 2. We used 24-h APF brains for proteomic analysis to capture the developmental window during which MB defects were observed in the absence of Nab2.

**Figure 3:**
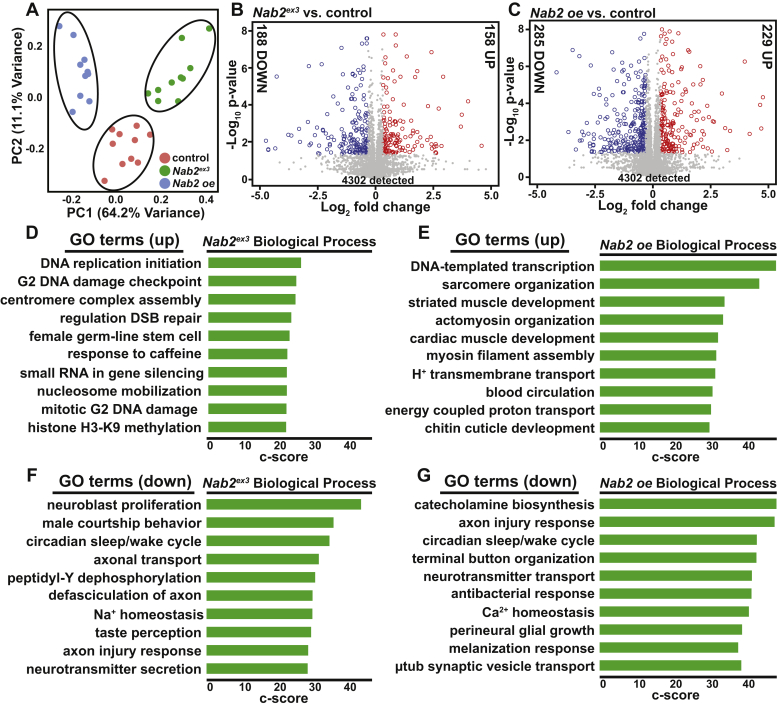
**Quantitative proteomic analysis of developmentally timed pupal brains reveals a role for Nab2 in neurodevelopment.***A*, principal component analysis (PCA) of proteomic data from 24 h after puparium formation *Drosophila* brains from ten biological replicates of *control*, *Nab2*^*ex3*^, and *Nab2 oe* flies (control = *C155>Gal4, w*^*1118*^*; Nab2*^*ex3*^ = *C155>Gal4;;Nab2*^*ex3*^; *Nab2 oe* =*C155>Gal4;Nab2*^*EP3716*^*;Nab2*^*ex3*^) show results cluster based on the genotype and that *Nab2*^*ex3*^ and *Nab2 oe* are distinct from control and each other. PCA was performed in RStudio using prcomp (default stats package v3.5.1), and summed peptide intensities were used as the input. *B* and *C*, volcano plots show proteins differentially expressed in each *Nab2* genotype compared with the control [*B*, *Nab2*^*ex3*^ (346; 188 down and 158 up) and *C*, *Nab2 oe* compared with the control (514; 285 down and 229 up)]. Ten biological replicates (n = 10) per genotype (20 brains per pooled biological replicate) with 30 technical replicates in total. Significance thresholds: log_2_(≥0.32 and ≤−0.32) and −log_10_(*p*-value) ≥1.3; thresholds were based on power calculation and instrumental limits. Protein abundance change (down or up) indicated on each side of the plot (log_2_*Nab2*^*ex3*^/Cont or *Nab2 oe*/Cont: *gray* = not significant, *blue* ≤ −0.32, *red* ≥ 0.32). The number of differentially expressed proteins to total detected proteins is shown atop the graph; 346 of 4302 *Nab2*^*ex3*^ proteins are differentially expressed (*B*) and 514 of 4302 *Nab2 oe* proteins are differentially expressed (*C*). *D*–*G*, the enriched gene ontology terms from FlyEnrichr database for biological process are shown for proteins increased log_2_(*Nab2*^*ex3*^/Cont) ≥0.32 in panel *D Nab2*^*ex3*^ and log_2_(*Nab2 oe*/Cont) in panel *E Nab2 oe* and decreased log_2_(*Nab2*^*ex3*^/Cont) ≤−0.32 in panel *F Nab2*^*ex3*^ and log_2_(*Nab2 oe*/Cont) in panel *G Nab2 oe*. The *bars* shown correspond to the top ten c-scores (c-score = ln(adj *p*-val) ∗ z-score) in each dataset (adjusted *p*-value <0.05) (74, 75, 76).

MS was carried out for ten biological replicates for each of the three genotypes (*control*, *Nab2*^*ex3*^, and *Nab2 oe*), with five male samples and five female samples analyzed separately. Across brain samples, a total of 4302 proteins were detected. Unbiased principal component analysis (PCA), which was performed using summed peptide intensities across all 30 samples per protein, per genotype, reveals three distinct clusters (Fig. 3*A*). The 30 plotted samples form three distinct clusters by genotype, indicating high similarity between male and female samples within a given genotype. Subsequent simple linear regression modeling of the data obtained indicated that male and female samples could be combined for analyses adding power. These combined datasets (n = 10 per genotype) were used for subsequent analyses.

### Proteomic analysis identifies proteins that change in abundance when Nab2 levels are altered

We first analyzed differences between each experimental genotype and control. Differentially expressed proteins were then identified for *Nab2*^*ex3*^ and *Nab2 oe* genotypes by comparing each to the control dataset (*Nab2*^*ex3*^ *versus control* and *Nab2 oe versus control*) with protein abundance change thresholds of log_2_(experimental/control) ≥0.32 or ≤−0.32 and a significance threshold of −log_10_(*p*-value) ≥1.3.

#### Nab2^ex3^*versus* control

Of the 4302 total proteins detected by LC-MS/MS across all three groups, 346 proteins (∼8% of total proteins detected) are differentially expressed in the *Nab2*^*ex3*^ brains *versus* control brains (Fig. 3*B*) (Table S1) (full dataset available at ProteomeXchange Consortium via PRIDE under the accession #PXD022984). Within this group, 158 proteins score ≥0.32 log_2_ fold change increase (five most elevated: CG1910, Got1, Ida, Mtp, and Wwox) and 188 proteins score ≤−0.32 log_2_ fold change decrease, with Nab2 among the top five most decreased (Nab2, Pglym78, Mkk4, Cortactin, and Psa) (Fig. 3*B*).

#### Nab2 oe *versus* control

Of 4302 total proteins detected, 514 proteins are differentially expressed in *Nab2 oe* brains relative to control brains (approximately 12% of total proteins detected). Within this group, 229 proteins score ≥0.32 log_2_ fold change increase (five most elevated: CG1910, Ccp84Ae, Ida, Ccp84Ag, and alien) and 285 proteins scored ≤−0.32 log_2_ fold change decrease (five most decreased: Pglym, Mkk4, cortactin, Gnmt, and CG34280) (Fig. 3*C*) (Table S1). Nab2 itself was the 32nd most elevated protein among the 229 proteins increased in abundance in *Nab2 oe* relative to control, confirming the effectiveness of the neuron-specific expression of the *C155>Gal4;Nab2*^*EP3716*^ genotype.

### Gene ontology analysis supports a role for Nab2 in neurodevelopment

Looking beyond individual protein changes can provide a broader understanding of the effects of disrupting Nab2. Therefore, gene ontology (GO) analysis for biological process enrichment was performed with FlyEnrichr by analyzing the differentially expressed (*Nab2*^*ex3*^ *versus control* and *Nab2 oe versus control*) protein datasets. This FlyEnrichr analysis reveals that proteins increased in the *Nab2*^*ex3*^ differentially expressed dataset represent biological processes involved in genome maintenance (*e.g.*, DNA replication initiation, G_2_ DNA damage checkpoint, centromere complex assembly) and development (*e.g.*, female germline stem cell) (Fig. 3*D*), while proteins increased in the *Nab2*^*oe*^ differentially expressed dataset represent processes related to development (*e.g.*, striated muscle development, cuticle development) and muscle organization (*e.g.*, sarcomere organization, myosin filament assembly) (Fig. 3*E*). Proteins decreased in the *Nab2*^*ex3*^ differentially expressed and *Nab2*^*oe*^ differentially expressed datasets are strongly enriched for processes linked to neurodevelopment, synaptic function, and brain maintenance (Fig. 3, *F* and *G*). Within the *Nab2*^*ex3*^ differentially expressed dataset, decreased proteins are enriched for the processes of neuroblast proliferation, circadian sleep/wake cycle, and axonal transport (Fig. 3*F*). Within the *Nab2*^*oe*^ differentially expressed dataset, decreased proteins are enriched for the processes of axon injury response, circadian sleep/wake cycle, and neurotransmitter transport (Fig. 3*G*).

**Figure 4:**
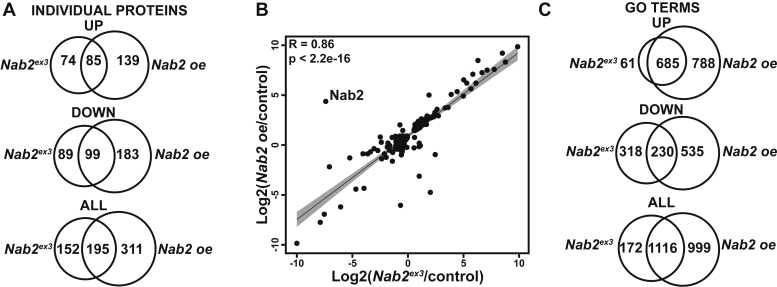
***Nab2***^***ex3***^**and *Nab2 oe* brains display distinct sets of differentially expressed proteins but have similar changes among shared proteins.***A*, Venn diagrams illustrating the number of individual, differentially expressed proteins, which are shared or unique to *Nab2*^*ex3*^ and *Nab2 oe* that (*top*) increase or (*middle*) decrease in protein abundance (*Nab2*^*ex3*^ relative to *control* and *Nab2 oe* relative to control) or (*bottom*) all abundance changes. *B*, a correlation curve comparing the changes in protein abundance for proteins changed in both *Nab2*^*ex3*^ and *Nab2 oe* relative to *control* was produced by plotting on a logarithmic scale. Results show that the shared changes (195) observed are highly correlated (R = 0.86, *p* < 2.2e-16, Pearson coefficient) in magnitude and direction. Regression line plotted in *black* with 95% confidence interval depicted by *gray shading*. Nab2 is expected to change in direction and magnitude between *Nab2*^*ex3*^ and *Nab2 oe* and is annotated on the plot. *C*, Venn diagrams illustrating the number of GO biological process terms enriched in *Nab2*^*ex3*^ and *Nab2 oe* that are shared or unique based on the subset of proteins that (*top*) increase or (*middle*) decrease protein abundance or (*bottom*) all abundance changes. GO, gene ontology.

Comparison of individual protein changes and FlyEnrichr GO terms between *Nab2*^*ex3*^ differentially expressed (346 proteins) and *Nab2*^*oe*^ differentially expressed (514 proteins) datasets provides some significant insights (Fig. 4, *A*–*C*). There are individual protein changes and GO terms that are shared between *Nab2*^*ex3*^-DE and *Nab2*^*oe*^-DE, and there are changes that are exclusive to one or the other dataset (Fig. 4*A*). Of the total differentially expressed proteins in both datasets, 23% are unique to *Nab2*^*ex3*^, 47% are unique to *Nab2 oe*, and 30% are shared between the two genotypes (referred to as “shared DE changes”) (Fig. 4*A*). Among the last category, in addition to protein identity, there is significant correlation in protein expression between *Nab2*^*ex3*^ and *Nab2 oe* shared DE changes (Fig. 4*B*). A total of 195 proteins accounted for the shared differentially expressed changes between *Nab2*^*ex3*^ and *Nab2 oe* brains (Fig. 4*A*), and these shared changes are highly correlated with one another (R = 0.86, *p* < 2.2^−16^; Fig. 4*B*). Of the 195 shared proteins, a large fraction (184 of 195, approximately 94%) changes abundance in *Nab2*^*ex3*^ differentially expressed and *Nab2*^*oe*^ differentially expressed datasets in the same direction (Fig. 4*B*). However, a subset of 11 shared differentially expressed proteins is altered in opposing directions, for example, increased in *Nab2*^*ex3*^ differentially expressed and decreased in *Nab2*^*oe*^ differentially expressed or vice versa (Table 1). Nab2 itself is one of these 11 shared proteins (Fig. 4*B*, Table 1). Nab2 is decreased relative to control in *Nab2*^*ex3*^ brains (log_2_(−8.36)) and increased relative to control in *Nab2 oe* brains (log_2_(3.94)) (Fig. 4*B*, Nab2-labeled data point). Finally, the *Nab2*^*ex3*^ differentially expressed and *Nab2*^*oe*^ differentially expressed datasets each have unique proteins that may provide insight into previously observed phenotypes in *Nab2* mutants or overexpression systems (15, 17, 20, 28, 37, 38). There are 152 proteins changed exclusively in *Nab2*^*ex3*^ brains relative to control, and 311 proteins changed exclusively in the *Nab2 oe* brains relative to control (Fig. 4*A*). As general overexpression of *Nab2* is more lethal than zygotic *Nab2* loss (15), the 311 changes unique to *Nab2 oe* may represent dominant effects of excess Nab2. However, the 152 proteins that are significantly changed only in *Nab2*^*ex3*^ brains, and not in the *Nab2 oe* genotype (which is in the *Nab2*^*ex3*^ background), are thus rescued by re-expression of WT Nab2 in *Nab2*^*ex3*^ brain neurons. These differences in *Nab2*^*ex3*^ and *Nab2 oe* differentially expressed proteins are also reflected in the FlyEnrichr GO analysis, which reveals 172 terms unique to *Nab2*^*ex3*^ and 999 unique to *Nab2 oe* (Fig. 4*C*). Differences between *Nab2*^*ex3*^ and *Nab2 oe* have the potential to provide insight into the neuroanatomical defects observed in *Nab2*^*ex3*^ pupal brains (Fig. 1*B*).

**Table 1:** *Nab2*^*ex3*^ and *Nab2 oe* shared proteins that change in different directions

Protein symbol	log_2_(*Nab2*^*ex3*^/Cont)	−log10(*p*-value)	Log_2_(*Nab2oe*/Cont)	−log10(*p*-value)
Nab2	−8.4	8.3	3.9	6.2
Hml	−1.1	2.0	1.0	3.3
Mhc	−0.8	1.8	0.3	4.0
LamC	0.3	2.2	−1.8	1.6
CG15369	0.4	2.3	−0.5	2.1
Sgs7	0.8	1.9	−1.7	2.2
Sgs5	0.8	3.1	−1.2	3.1
Sgs3	0.8	1.3	−5.3	2.7
Sgs8	0.9	2.2	−1.5	3.0
Eig71Ed	1.9	2.2	−7.3	2.0
Sls	2.4	6.8	−2.6	1.3

**Figure 5:**
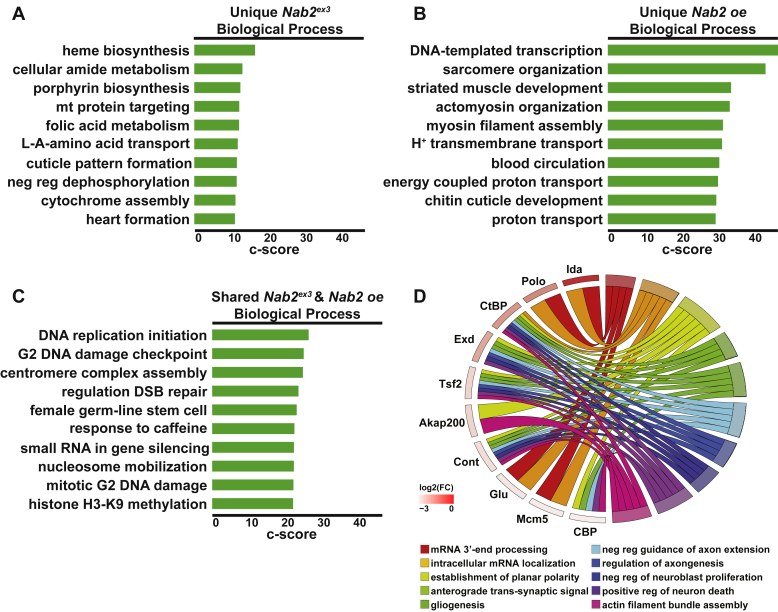
**Proteins increased in abundance in *Nab2***^***ex3***^**and *Nab2 oe* brains are enriched for processes including RNA processing and neurodevelopment.***A*–*C*, the enriched terms from FlyEnrichr database for biological process resulting from the subset of proteins increased in abundance that are (*A*) unique to *Nab2*^*ex3*^, (*B*) unique to *Nab2 oe*, and (*C*) shared between *Nab2*^*ex3*^ and *Nab2 oe*. The *bars* shown correspond to the top ten c-scores (c-score = ln(adj *p*-val) ∗ z-score) in each dataset (adj. *p*-val < 0.05) (74, 75, 76). *D*, a chord plot showing how proteins are represented in multiple GO biological process terms enriched from the subset of proteins increased in abundance in both *Nab2*^*ex3*^ and *Nab2 oe* relative to control. The selected terms are shown on the *right* of the plot and are color-coded according to the legend, with the chords extending to the *left* of the plot showing which proteins are represented in each term. The log_2_ (*Nab2*^*ex3*^/Cont) in *Nab2*^*ex3*^ is represented by color change (*white* to *red*) next to each protein annotation. GO, gene ontology.

As previous studies suggest Nab2 can function as a translational repressor (20, 21), the most direct Nab2 targets could be expected to increase in abundance upon loss of Nab2 function (*Nab2*^*ex3*^). However, factors that decrease in protein abundance, whether due to direct or indirect effects of Nab2, may also be phenotypically significant in the *Nab2*^*ex3*^ genotype. To parse these effects, the unique and shared changes in the *Nab2*^*ex3*^-DE and *Nab2*^*oe*^-DE datasets were further divided into increased and decreased groups, and then subjected to FlyEnrichr analysis (Fig. 4*C*). Protein increases unique to the *Nab2*^*ex3*^ differentially expressed dataset represent processes involved in metabolism (Fig. 5*A*), while increases unique to the *Nab2*^*oe*^ differentially expressed dataset represent processes involved in tissue development and organization (Fig. 5*B*). The increases common to both *Nab2*^*ex3*^ differentially expressed and *Nab2*^*oe*^ differentially expressed datasets are enriched in processes involved in genome maintenance and development (Fig. 5*C*). A chord plot of biological process GO terms relating to RNA processing and neurodevelopment highlights proteins *increased* in both datasets (Fig. 5*D*). Among these are the glial-neuronal adhesion protein contactin, the planar cell polarity (PCP) accessory protein A-kinase anchor protein 200, the condensin subunit gluon, and the neuroblast regulator Polo (Fig. 5*D*).

**Figure 6:**
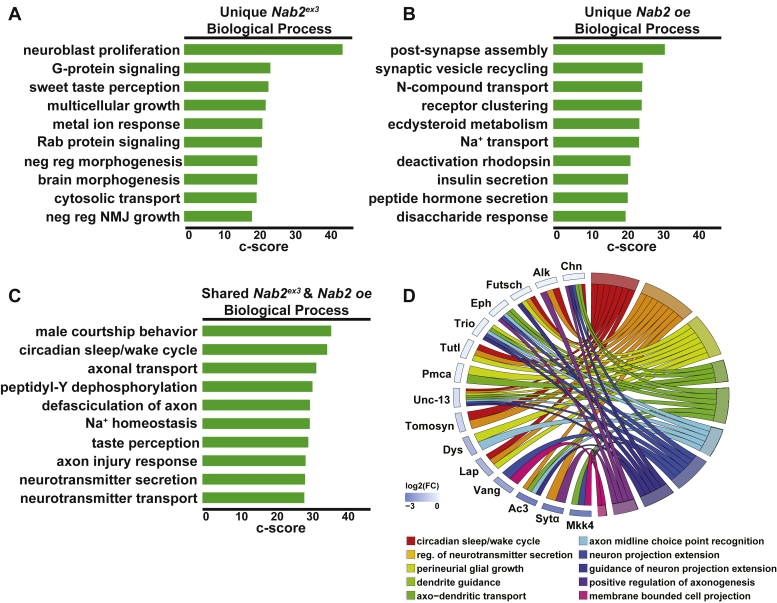
**Proteins reduced in abundance in *Nab2***^***ex3***^**and *Nab2 oe* are enriched for neurological roles.***A*–*C*, the enriched terms from FlyEnrichr database for biological process resulting from the subset of proteins decreased in abundance that are (*A*) unique to *Nab2*^*ex3*^, (*B*) unique to *Nab2 oe*, and (*C*) shared between *Nab2*^*ex3*^ and *Nab2 oe*. The *bars* shown correspond to the top ten c-scores (c-score = ln(adj *p*-val) ∗ z-score) in each dataset (adj. *p*-val < 0.05) (74, 75, 76). *D*, a chord plot showing how proteins are represented in multiple GO biological process terms enriched from the subset of proteins decreased in abundance in both *Nab2*^*ex3*^ and *Nab2 oe* relative to control. The selected terms are shown on the *right* of the plot and color-coded according to the legend, with the chords extending to the *left* of the plot showing which proteins are represented in each term. The log_2_(*Nab2*^*ex3*^/Cont) in *Nab2*^*ex3*^ is represented by color change (*white* to *blue*) next to each protein annotation. GO, gene ontology.

A similar analysis of shared and exclusive decreased proteins between the *Nab2*^*ex3*^ differentially expressed and *Nab2*^*oe*^ differentially expressed datasets (Fig. 6, *A*–*D*) reveals that decreases unique to *Nab2*^*ex3*^ are enriched for the processes of neuroblast proliferation, taste perception, and brain morphogenesis (Fig. 6*A*), while unique *Nab2 oe* decreases are enriched for the processes of postsynapse assembly, synaptic vesicle recycling, and sodium ion transport (Fig. 6*B*). The shared decreases between *Nab2*^*ex3*^ and *Nab2 oe* represent processes involved in neurodevelopment and brain function (Fig. 6*C*). A chord plot of biological process GO terms relating to neurodevelopment, behavior, and brain function highlights proteins decreased in both datasets (Fig. 6*D*). Among these are the microtubule-associated protein Futsch, the neuronal Ig-family transmembrane protein turtle, the axon guidance and PCP component Vang, and the Rho GEF Trio (Fig. 6*D*). The proteomic changes revealed here resulting from disruption of the RBP Nab2 likely correspond in part to changes in mRNA regulation.

### Shared protein changes between Nab2^ex3^ flies and ZC3H14^*Δex13/Δex13*^ mice

**Figure 7:**
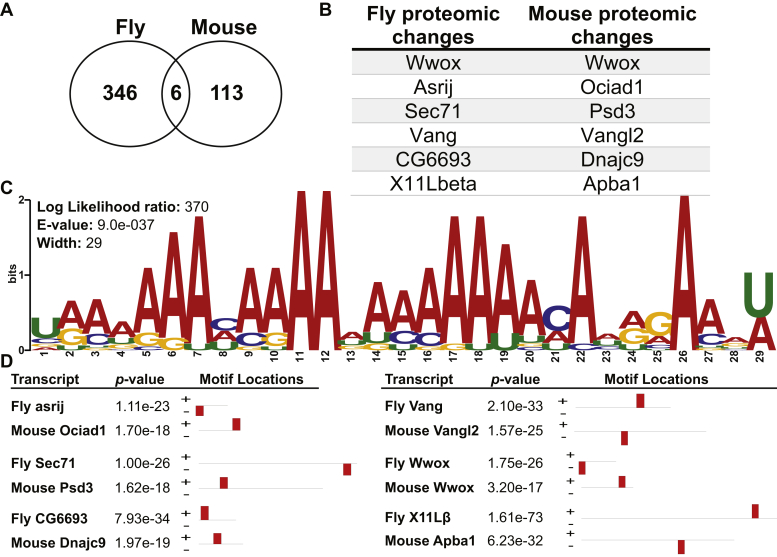
**Six protein changes are shared between Nab2**^**ex3**^**flies and ZC3H14**^***Δex13/Δex13***^**mice and contain a shared A-rich motif.***A*, Venn diagram showing the total number of differentially expressed proteins in *Nab2*^*ex3*^ pupal brain (346 proteins) and *Zc3h14*^*Δex13/Δex13*^ P0 hippocampi (113 protein) (21) with six shared protein changes. *B*, list showing the six shared differentially expressed proteins between *Nab2*^*ex3*^ pupal brain and *Zc3h14*^*Δex13/Δex13*^ P0 hippocampi. This study found proteomic changes (log_2_(*Nab2*^*ex3*^/Cont)) of Wwox log_2_(7.5); Asrij log_2_(0.6); Sec71 log_2_(−1.3); Vang log_2_(−2.3); CG6693 log_2_(−2.8); and X11Lβ log_2_(−4.7). *C*, MEME logo of A-rich motif identified in the 12 transcripts encoding the six fly proteins and the six mouse proteins. MEME conducted with OOPS (exactly one site per sequence) motif site distribution, with minimum motif width of six and maximum motif width of 50. Analysis performed under MEME version 5.3.2 (release date: 02/06/2021) (39, 40). This motif was the most enriched among the transcripts with a log likelihood ratio of 370, E-value of 9.0e-37, and width of 29. Threshold of significance: E-value <0.05. *D*, A-rich motif location shown within the transcripts corresponding to the differentially expressed proteins. Fly and mouse transcript pairs are shown with transcript name, the *p*-value significance of motif, and motif location within the transcript (indicated by *red bars*). MEME, Multiple Expectation maximizations for Motif Elicitation.

Comparing the differentially expressed proteins from *Nab2*^*ex3*^ brains to a previously reported proteomic dataset generated from the hippocampi of P0 *Zc3h14* KO (*Zc3h14*^*Δex13/Δex13*^) mice (21) reveals six proteomic changes shared between flies and mice (Fig. 7, *A* and *B*). These conserved changes may give insight into conserved targets of Nab2/ZC3H14. The transcripts, of these conserved protein changes, may represent targets of Nab2/ZC3H14 and thus may share a sequence motif recognized by Nab2/ZC3H14. To test for shared motifs among this set of conserved candidate target RNAs, sequence analysis was performed using Multiple Expectation maximizations for Motif Elicitation (MEME) (39, 40). The transcripts representing the 12 shared proteins, six from flies and six from mice, were used as input for MEME analysis (Fig. 7, *A* and *B*). MEME discovers novel, ungapped motifs and identified a 29-bp-long, internal-A-rich motif as the most enriched among the transcripts (Fig. 7*C*). This 29-bp motif (log likelihood ratio 370, E-value 9.0e-37) is overrepresented in these transcripts relative to the random chance expected across the transcriptome. The shared motif across these conserved targets suggests this could be a binding sequence common to fly Nab2 and mouse ZC3H14. The location of this 29-bp motif varies among the transcripts analyzed (Fig. 7*D*, Fig. S1).

## Discussion

Here, we examine the role of a conserved RBP in neurodevelopment by exploiting a *Drosophila* model. Using carefully timed brain collections, we find that axon projection and development of MB α- and β-lobes structure are severely perturbed in pupal brains, and that coincident with these defects in axonal trajectories, we detect clear changes in a small fraction (∼8%) of the brain proteome. This restricted effect on a subset of brain proteins is consistent with our recent finding that Nab2 loss has specific effects on the brain transcriptome (28) and supports the hypothesis that Nab2 regulates expression of a subset of neuronal mRNAs and proteins that are involved in various neurodevelopmental processes, including axon growth and guidance in the MBs.

Bioinformatic analysis of differentially expressed proteins in *Nab2*^*ex3*^ mutant brains relative to control samples indicates that Nab2-regulated proteins are enriched in functional classes corresponding to axonal development but also suggest a potential role in dendrites. The former link to axonogenesis matches the observed MB α- and β-lobe defects, but the latter link to dendritic proteins is more novel and may be conserved. The murine Nab2 homolog, ZC3H14, localizes to dendritic shafts and spines and controls dendritic spine morphology in cultured neurons (21, 41). Nab2-regulated proteins identified in the present study that have predicted dendritic roles include the PCP factor Vang, the adhesion protein cortactin, the netrin receptor frazzled, the neuronal Ig-family transmembrane protein turtle, the fragile-X mental retardation homolog Fmr1, the Rho GEF trio, the RBP Alan Shepherd/RBMS3, and the microtubule-associated protein Futsch. Significantly, a proteomic dataset generated from the hippocampi of P0 *Zc3h14* KO mice (21) also shows enrichment for the Vang homolog Vang like-2, in addition to five other neurodevelopmental proteins that are also detected here as differentially expressed in *Nab2*^*ex3*^ pupal brains: the oxioreductase Wwox, the PDZ-domain protein X11Lβ/Apba1, the DnaJ protein CG6693/Dnajc9, the ARF-GEF factor Sec71/Psd3, and the endosomal protein Asrij/Ociad1 (Table 1).

Human ZC3H14 expressed in neurons of *Nab2*^*ex3*^ flies rescues many of the Nab2 null phenotypes (15). This finding suggests that there should be shared function and RNA targets between mammalian ZC3H14 and fly Nab2. The 29-bp, A-rich motif identified in the transcripts represented by these conserved protein changes between flies and mice (Fig. 7*C*) may represent a target binding motif for Nab2/ZC3H14. The potential for this A-rich motif to be a Nab2-binding site is supported by the previous definition of a Nab2-binding motif in *Saccharomyces cerevisiae* (A11G and A12) (19, 42, 43). This A-rich motif identified in the present study by examining conserved proteomics changes between *Nab2*^*ex3*^ fly brains and *ZC3H14*^*Δex13/Δex13*^ mouse hippocampi is similar to a recently identified A-rich motif defined via RNA-IP of fly Nab2 (38).

The evidence to suggest conserved target RNAs suggests that Nab2/ZC3H14 may have a shared role in regulating key RNAs involved in neuronal development and signaling. Of note, fly Nab2 physically and functionally interacts with the *Drosophila* Fragile-X mental retardation protein (Fmr1) (20), which has a key role in postsynaptic, activity-dependent local mRNA translation and is required for normal dendritic morphology (13).

Our comparison of the effects of Nab2 dosage reveals that almost one-third of proteomic changes (29%) that occur in Nab2-deficient pupal brains are shared in brains with neuronal overexpression of Nab2. Of 195 proteins that change in abundance in the *Nab2*^*ex3*^ and *Nab2 oe* datasets, only 11 of these are inverse changes (*i.e.*, increased in *Nab2*^*ex3*^ and decreased in *Nab2 oe* or vice versa) while the other 184 proteins change in the same direction between these two genotypes (*i.e.*, increased or decreased in both *Nab2*^*ex3*^ and *Nab2 oe*). A simplistic model would predict that loss and gain of Nab2 would have the opposite effect on targets, but these data suggest that excess Nab2 can generate a dominant-negative effect on some candidate target RNAs, perhaps by sequestering Nab2-interacting proteins or blocking access of other RBPs to sites on RNAs. The 184 shared protein changes that occur in the same direction can be explained either by a dominant-negative effect of Nab2 overexpression or by the nature of the experiment where the *Nab2 oe* is performed in a background of *Nab2 ex3* flies. As the *Nab2 ex3* is a zygotic allele (15) and *Nab2 oe* is driven by a neuron-specific promoter (*C155>Gal4*), the shared proteomic changes could reflect changes in non-neuronal cell types. Indeed, the 11 proteins that show inverse changes in the *Nab2*^*ex3*^ and *Nab2 oe* datasets could represent a subset of targets that respond in a linear fashion to Nab2 dose in neurons. One possibility is that the mRNAs encoding these proteins represent direct targets of the Nab2 RBP. Our analysis detects 152 significantly changed proteins in *Nab2*^*ex3*^ brains that are rescued back to normal levels in *Nab2 oe* brains, which parallels the morphological rescue of *Nab2*^*ex3*^ by *Nab2 oe* documented in prior studies (15, 16, 17). Among the proteins in this group is tumbleweed, which is homologous to human RacGap1 and required for normal MB development (44). This putative link from Nab2 to tumbleweed-based control of MB patterning warrants further study.

Evidence of interactions between *Nab2* and elements of the miRNA machinery (*e.g.*, argonaute) and ncRNA-processing factors (*e.g.*, *Rm62*) detected in our prior work (17, 20) are also supported by these proteomic analyses. Seven GO terms relating to miRNA/ncRNA are enriched in the *Nab2*^*ex3*^ dataset including pre-miRNA processing, production of small RNA involved in gene silencing by RNA, and ncRNA 3’-end processing. As miRNAs and ncRNAs can regulate gene expression (45), some observed effects of *Nab2* alleles on the brain proteome could be indirect, rather than changes to direct (*i.e.*, bound) Nab2 target RNAs. This model aligns with our prior work showing that Nab2 physically associates with Fmr1 and coregulates some mRNAs (20). In the adult brain, depletion of Nab2 derepresses a *CamKII* translation but Nab2 depletion has no effect on *futsch* (20). In the present study of pupal brains, Futsch protein is decreased in *Nab2*^*ex3*^ brains (log_2_(*Nab2*^*ex3*^/contactin) = −0.38), whereas CamKII protein levels are not significantly changed. These stage-specific effects on the brain proteome raise the possibility that Nab2 interactions are not only target-specific (*e.g.*, as in the case of alternative splicing) (28) but can also vary across developmental stages.

As noted above, the PCP component Vang and the Vang murine homolog Vang like-2 are among a small group of proteins that are differentially expressed in both *Drosophila Nab2*^*ex3*^ pupal brains and in P0 hippocampi dissected from *Zc3h14* KO mice (21) (Table 1). This finding is particularly significant, given the strong genetic interactions detected between an eye-specific *Nab2* overexpression system (*GMR-Nab2*) and multiple PCP alleles, including an allele of *Vang* (37). The PCP pathway plays a conserved role in regulating axon projection and guidance in multiple higher eukaryotic species (46, 47, 48, 49, 50), including in the *Drosophila* MBs (51, 52, 53, 54, 55). Thus, the change in levels of Vang, a core PCP component (56, 57, 58, 59, 60), in *Nab2*^*ex3*^ brains could provide an additional, direct link from Nab2 to a pathway that guides neurodevelopment including the MB α- and β-lobes.

In aggregate, these data provide a comprehensive view of the role Nab2 plays in regulating abundance of a specific cohort of proteins in the developing pupal brain, some of which are likely to correspond to mRNAs that are bound and regulated by Nab2 in brain neurons. Furthermore, this set of proteins is enriched for neurodevelopmental factors that could represent evolutionarily conserved targets of this class of zinc finger RBPs.

## Experimental procedures

### *Drosophila* genetics

All crosses were maintained in humidified incubators at 25 °C with 12-h light–dark cycles unless otherwise noted. The *Nab2*^*ex3*^ loss-of-function mutant has been described previously (15). Alleles and transgenes: *Nab2*^*EP3716*^ (Bloomington (BL) #17159) and *P{GawB}elav*^*C155*^ (BL #458), and *w*^*1118*^ (‘*control’*; BL #3605).

### Brain imaging, statistical analysis, and visualization

Brain dissections were performed as previously described (17). Briefly, 48 to 72 h APF, brains were dissected in PBS (1xPBS) at 4 °C, fixed in 4% paraformaldehyde at RT, washed 3× in PBS, and permeabilized in 0.3% PBS-T (1xPBS and 0.3% Triton X-100). After blocking for 1 h (0.1% PBS-T, 5% normal goat serum), brains were stained overnight in block + primary antibodies. After 5× washes in PBS-T, brains were incubated in block for 1 h, moved into block + secondary antibody for 3 h, and then washed 5× in PBS-T and mounted in VECTASHIELD (Vector Labs). The anti-FasII monoclonal antibody 1D4 (Developmental Studies Hybridoma Bank) was used at 1:20 dilution. Whole brain anti-FasII images were captured on a Nikon AR1 HD25 confocal microscope using NIS-Elements C Imaging software v5.20.01, and maximum intensity projections were generated in ImageJ Fiji. MB morphological defects were called as α-lobe thinning or missing and β-lobe fusion or missing for *control* (α-lobe = 11 biological and 22 technical replicates; β-lobe = 11 biological and technical replicates) and *Nab2*^*ex3*^ (α-lobe = 17 biological and 34 technical replicates; β-lobe = 17 biological and technical replicates). Quantitation of MB phenotypes was performed as previously described (17).

### Global proteomics

#### Sample collection

Five biological replicates of control, *Nab2*^*ex3*^, and *Nab2 oe* for both female and male brains were collected at 23.25 to 25.5 h APF (five pools per condition, 20 brains per pool), lysed in urea buffer (8 M urea, 100 mM NaHPO_4_, pH 8.5) with HALT protease and phosphatase inhibitor (Pierce) and processed at the Emory Proteomics Core.

#### LC-MS/MS

Data acquisition by LC-MS/MS was adapted from a previously published procedure (61). Derived peptides were resuspended in 20 μl of the loading buffer (0.1% TFA). Peptide mixtures (2 μl) were separated on a self-packed C18 (1.9 μm, Dr Maisch) fused silica column (25 cm × 75 μM internal diameter; New Objective) and were monitored on an Orbitrap Fusion Tribrid Mass Spectrometer (Thermo Fisher Scientific). Samples were run in 30 technical replicates of five biological replicates per condition. Elution was performed over a 130-min gradient at 250 nl/min with buffer B ranging from 3% to 99% (buffer A: 0.1% formic acid in water, buffer B: 0.1% formic acid in acetonitrile). The mass spectrometer duty cycle was programmed to collect at top speed with 3-s cycles. The full MS scans (300–1500 m/z range, 50-ms maximum injection time) were collected at a nominal resolution of 120,000 at 200 m/z and automatic gain control target of 200,000 ion counts in the profile mode. Subsequently, the most intense ions above an intensity threshold of 5000 were selected for higher-energy collision dissociation (0.7 m/z isolation window with no offset, 30% collision energy, 10,000 automatic gain control target, and 35-ms maximum injection time), and the MS/MS spectra were acquired in the ion trap. Dynamic exclusion was set to exclude previously sequenced precursor ions for 30 s within a 10-ppm window. Precursor ions with charge states 2 to 7 were included.

#### MaxQuant protein identification

Label-free quantification analysis was adapted from a previously published procedure (62). Data files for the samples were analyzed using MaxQuant v1.5.2.8 with Thermo Foundation 2.0 for RAW file reading capability. Spectra were searched using the search engine Andromeda and integrated into MaxQuant against the *Drosophila melanogaster* UniProt database (43,836 target sequences, downloaded in February, 2018). The Andromeda score measures how an acquired spectrum matches the theoretical fragment masses and is defined as the −10 logarithmic probability of observing the given number of matches or more by chance (63). Methionine oxidation (+15.9949 Da), asparagine and glutamine deamidation (+0.9840 Da), and protein N-terminal acetylation (+42.0106 Da) were variable modifications (up to five allowed per peptide); cysteine was assigned as a fixed carbamidomethyl modification (+57.0215 Da). Only fully tryptic peptides were considered with up to two missed cleavages in the database search. A precursor mass tolerance of ±20 ppm was applied before mass accuracy calibration and ±4.5 ppm after internal MaxQuant calibration. Other search settings included a maximum peptide mass of 6000 Da, a minimum peptide length of six residues, 0.6 Da tolerance for ion trap MS/MS scans. Cofragmented peptide search was enabled to deconvolute multiplex spectra. The false discovery rates for peptide spectral matches, proteins, and site decoy fraction were all set to 1%. Quantification settings were as follows: requantify with a second peak finding attempt after protein identification has completed; match MS1 peaks between runs; a 0.7-min retention time match window was used after an alignment function was found with a 20-min RT search space. Quantitation of proteins was performed using summed peptide intensities given by MaxQuant. The quantitation method only considered razor plus unique peptides for protein level quantitation.

### Statistical analysis and data visualization

Statistical analyses were performed in either RStudio v1.1.453 or GraphPad Prism 8. Statistical analyses for MB phenotypes and plotting were performed using GraphPad. Significance was determined using Student’s *t* test. Graphs reported either quartile ranks or error bars representing SD. Significance scores indicated on graphs are ∗*p* ≤ 0.05, ∗∗*p* ≤ 0.01, and ∗∗∗*p* ≤ 0.001. Statistical analyses for the proteomics, including differential expression analysis, linear regression modeling, and comparison across genotypes of protein and GO term differences were performed using RStudio v1.1.453 (64), custom in-house scripts, and the following packages: ggpubr v0.2 (65), cluster v2.1.0 (66), and GOplot v1.0.2 (67). Five biological replicates of control, *Nab2*^*ex3*^, and *Nab2 oe* for both female and male brains were collected as five pools per condition with 20 brains per pool (each pool meets the needed amount of protein for detection on Orbitrap Fusion Tribrid mass spectrometer). Simple linear regression modeling was performed to test variability across biological replicates including covariates of the genotype, sample ID, and sex. The results of the models did not support the null hypothesis that the LFQ value of the biological replicates was dependent on sample ID (F = 0.0888) or sex (F = 0.2135). Linear modeling was performed in RStudio using lm (default stats package v3.5.1) (68, 69). Based on modeling results, no samples were removed, but male and female samples were combined based on the genotype (n = 10 per genotype). Subsequent analyses consist of ten biological replicates per genotype (20 brains per pooled biological replicate) with 30 technical replicates in total. By applying Benjamini–Hochberg false discovery rate correction to group-wise ANOVA *p*-values, significant differentially expressed proteins were determined. Thresholds for significance of differentially expressed proteins were set at log_2_(protein abundance change genotype 1/protein abundance change genotype 2) ≥0.32 or ≤−0.32 and −log_10_(*p*-value) ≥1.3 (equivalent to individual protein adj *p*-val < 0.05), which were based on power calculation and instrumental detection limits. Protein abundance ratios use LFQ values. In addition, for quality control, all proteins with fewer than eight peptide reads were not considered for further analysis. PCA was performed in RStudio using prcomp (default stats package v3.5.1) and summed peptide intensities were used as input (70, 71, 72, 73). Input data came from 24-h APF *Drosophila* brains from ten biological replicates of *control*, *Nab2*^*ex3*^, and *Nab2 oe* flies (*control* = *C155>Gal4, w*^*1118*^*; Nab2*^*ex3*^ = *C155>Gal4;;Nab2*^*ex3*^; *Nab2 oe* = *C155>Gal4;Nab2*^*EP3716*^*;Nab2*^*ex3*^). Prcomp PCA was conducted (k = 3) with mapping of normal confidence ellipses and post hoc genotype labeling. Ellipses indicate significance of clusters; Prcomp default ellipse assumes a multivariate t-distribution. GO analyses were performed using FlyEnrichr (FlyEnrichr:amp.pharm.mssm.edu/FlyEnrichr/; accessed June 2020) (74, 75, 76). FlyEnrichr is a *Drosophila*-specific GO enrichment analysis package. Input data were differentially expressed proteins (*Nab2*^*ex3*^ relative to *control*; *Nab2 oe* relative to *control*). FlyEnrichr analyses were performed under default conditions with following term databases used: Coexpression Predicted GO Biological Process 2018, GO Biological Process AutoRIF Predicted zscore, and GO Biological Process AutoRIF. Significance of terms were determined using c-scores (c-score = ln(adj *p*-val) ∗ z-score) in each dataset and a threshold of adjusted *p*-value <0.05. C-score is the combined score of the *p*-value computed using Fisher’s exact test and the z-score computed to assess the deviation from the expected rank (74, 75, 76). FlyEnrichr corrects for multiple hypotheses using the Benjamini–Hochberg procedure with a threshold of 0.05. MEME analysis conducted with OOPS (exactly one site per sequence) motif site distribution, with minimum motif width of six and maximum motif width of 50. Threshold of significance: E-value < 0.05. E-value estimates the number of motifs, given the log likelihood ratio, accounting for the width and site count, that one would find in a set of random sequences. Where appropriate, additional analysis parameters used default settings. The analysis was performed under MEME version 5.3.2 (release date: 02/06/2021) (39, 40).

## Data availability

The mass spectrometry proteomics data have been deposited to the ProteomeXchange Consortium via the PRIDE (77) partner repository with the dataset identifier PXD022984. All remaining data are contained within the article.

## Supporting information

This article contains supporting information (39, 40).

## Conflict of interest

The authors declare that they have no conflicts of interest with the contents of this article.
