# Supplementary material for: The RNA-binding protein Nab2 regulates the proteome of the developing Drosophila brain
Source: J Biol Chem. 2021 Jun 15;297(1):100877. doi: 10.1016/j.jbc.2021.100877 (PMC8260979; doi:10.1016/j.jbc.2021.100877)
Supplement: Supplemental Figure S1 [file mmc1.pdf]

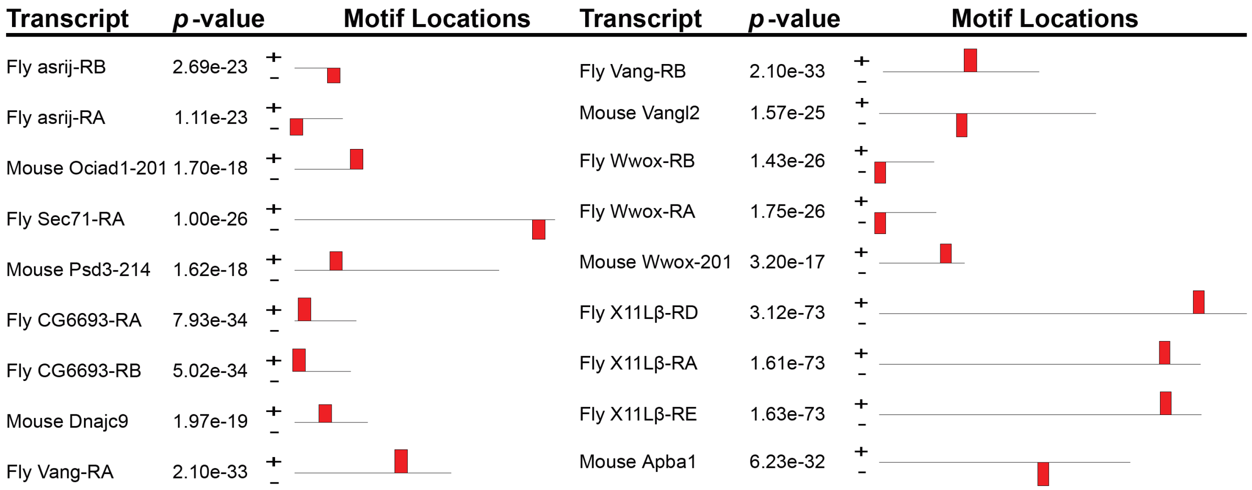

Supplementary Figure 1. A novel A-rich motif is shared among all transcripts corresponding to shared protein changes shared between Nab2^{ex3} flies and ZC3H14$^{\Delta ex13/\Delta ex13}$ mice. (A) Novel A-rich motif location shown with the 18 transcripts corresponding to the differentially expressed proteins. Fly and mouse transcript pairs are shown with transcript name, the p-value significance of motif, and motif location within the transcript (indicated by **red** bar). MEME (Multiple EM for Motif Elicitation) conducted with OOPS (exactly one site per sequence) motif site distribution, with minimum motif width of six and maximum motif width of fifty. Analysis performed under MEME version 5.3.2 (release date: 02/06/2021) (40, 41). Threshold of significance: p-value <0.05.
